# Evolution of Extra-Nigral Damage Predicts Behavioural Deficits in a Rat Proteasome Inhibitor Model of Parkinson's Disease

**DOI:** 10.1371/journal.pone.0017269

**Published:** 2011-02-25

**Authors:** Anthony C. Vernon, William R. Crum, Saga M. Johansson, Michel Modo

**Affiliations:** 1 Department of Neuroscience, Centre for the Cellular Basis of Behaviour, Institute of Psychiatry, Kings College London, London, United Kingdom; 2 Department of Neuroimaging, Institute of Psychiatry, Kings College London, London, United Kingdom; National Institutes of Health, United States of America

## Abstract

Establishing the neurological basis of behavioural dysfunction is key to provide a better understanding of Parkinson's disease (PD) and facilitate development of effective novel therapies. For this, the relationships between longitudinal structural brain changes associated with motor behaviour were determined in a rat model of PD and validated by post-mortem immunohistochemistry. Rats bearing a nigrostriatal lesion induced by infusion of the proteasome inhibitor lactacystin into the left-medial forebrain bundle and saline-injected controls underwent magnetic resonance imaging (MRI) at baseline (prior to surgery) and 1, 3 and 5 weeks post-surgery with concomitant motor assessments consisting of forelimb grip strength, accelerating rotarod, and apormorphine-induced rotation. Lactacystin-injected rats developed early motor deficits alongside decreased ipsilateral cortical volumes, specifically thinning of the primary motor (M1) and somatosensory cortices and lateral ventricle hypertrophy (as determined by manual segmentation and deformation-based morphometry). Although sustained, motor dysfunction and nigrostriatal damage were maximal by 1 week post-surgery. Additional volume decreases in the ipsilateral ventral midbrain; corpus striatum and thalamus were only evident by week 3 and 5. Whilst cortical MRI volume changes best predicted the degree of motor impairment, post-mortem tyrosine hydroxylase immunoreactivity in the striatum was a better predictor of motor behaviour overall, with the notable exception of performance in the accelerating rotarod, in which, M1 cortical thickness remained the best predictor. These results highlight the importance of identifying extra-nigral regions of damage that impact on behavioural dysfunction from damage to the nigrostriatal system.

## Introduction

Region-specific loss of dopaminergic neurons in the substantia nigra pars compacta (SNc) is the pathological hallmark of Parkinson's disease (PD), a progressive neurodegenerative movement disorder [1]. Neuronal loss is accompanied by formation of intraneuronal inclusions, Lewy bodies, composed primarily of the protein α-synuclein [2]. The anatomical and functional changes in PD may be classified into a three phase model: (1) mesenchephalic (dopaminergic neuronal loss), (2) basal ganglia (dopaminergic deafferentation) and (3) cortical (functional reorganisation) [3]. Longitudinal investigations using structural magnetic resonance imaging (MRI) provide a framework to map the sequence of neuroanatomical changes at all levels of this model. This is an advantage over techniques such as positron emission tomography (PET), which can only focus on one level, for example pre-synaptic dopamine terminals [4]. This information may then be related to clinical symptoms in patients to identify their neuroanatomical causes. This approach has been used successfully in several clinical studies [3], [5], [6], [7], [8], but such studies are lacking in animal models. MRI is well suited for this purpose, since the high anatomical resolution permits collection of quantitative information on morphological changes in the brains of disease models, which may be directly correlated with behavioural phenotypes [9], [10], [11]. Notably, such studies in animal models offer a significant advantage in that the neuropathology underlying MRI signal changes may be investigated. Moreover, this approach has the potential to identify surrogate markers of disease progression, which may be beneficial in the evaluation of novel pre-clinical models of PD and evaluation of experimental PD therapeutics [9], [10], [12].

Although several MRI studies have been conducted in both primate [13], [14], [15] and rodent models of PD [16], [17], [18], [19], [20], [21], these have primarily focussed exclusively on the nigrostriatal system or on changes in brain function, rather than structure. Previously, we have identified *in vivo* a pattern of morphological changes in several brain regions in rats lesioned by intranigral injection of the proteasome inhibitor lactacystin, which were associated with behavioural impairment in this model [11]. Intracranial injection of proteasome inhibitors into the nigrostriatal system is a model system that recapitulates key features of PD, including α-synuclein aggregation [11], [22], [23], [24], [25]. However, whilst dopaminergic neurons may be preferentially sensitive to proteasome inhibition [26], synthetic proteasome inhibitors may also induce non-specific neuronal toxicity [27], [28] and affect astrocyte proliferation and morphology [29], [30]. Thus, the aim of the current study was to map the evolution of neurodegenerative changes (primary and secondary) in the lactacystin model and examine their relevance to behavioural dysfunction using a combination of MRI, behavioural assessment and linear regression analysis. Post-mortem analyses of the brain were also conducted to identify potential neurobiological substrate(s) underlying *in vivo* morphometric changes. We hypothesized that structural brain changes in the extra-nigral regions, as well as the nigrostriatal system, underlie motor behavioural impairment *in vivo*.

## Materials and Methods

### Experimental animals

Male Sprague-Dawley rats (250±10g, Harlan, UK) were housed in groups of three at 21±1°C on a 12 hr light∶ dark cycle (lights on 0700, lights off 1900). Standard rat chow and drinking water were available *ad libitum*. All animal experiments were carried out in accordance with the Home Office Animals (Scientific procedures) Act, UK, 1986 and were approved by the Kings College London ethical review panel (Designation no. PCD 70/2901.)

### Experimental design

**Figure 1:**
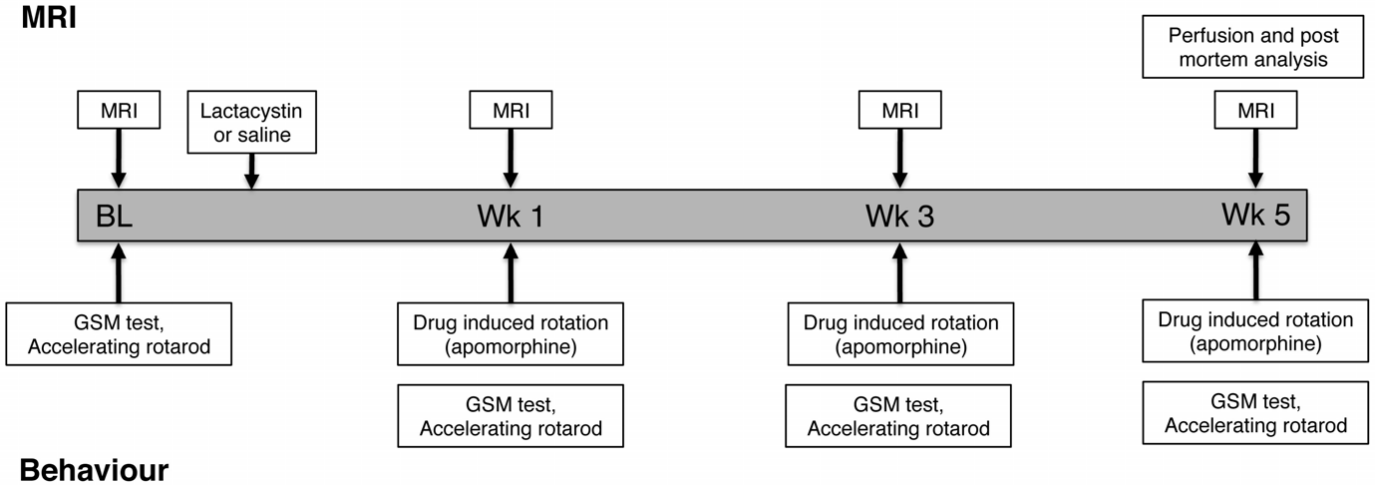
Experimental design and time-points for MRI and behavioural analysis in animals undergoing longitudinal examination. Additional cohorts of lesioned and control animals were sacrificed at weeks 1 and 3, respectively, for histological analysis.

The experimental design for this study is shown in Figure 1. Animals were randomly assigned to either control or lesion groups using a random number sequence (lesion *N* = 8; control *N* = 7). All animals underwent serial evaluation of brain structure by MRI and motor behavioural testing at baseline prior to surgery and 1, 3 and 5 weeks (wks) post-surgery (Figure 1). Drug-induced rotation as an index of nigrostriatal damage was assessed at 1, 3 and 5 wks post-surgery. All animals underwent post-mortem histological assessment. Two separate cohorts of animals were also lesioned with lactacystin or saline, but sacrificed at 1 or 3 wks post-lesion respectively. These animals were included for comparative analysis of post-mortem neuropathological changes at these time-points to investigate the pathology underlying MRI signal changes at 1 and 3 wks *in vivo*.

### Induction of nigrostriatal lesions

Nigrostriatal lesions were induced as previously described, with saline and lactacystin designated animals operated on in a randomised fashion in the same surgical session. [11]. Briefly, animals were anaesthetised by i.p. injection of a mixture of medetomidine hydrochloride (Domitor®, 0.25 mg/kg) and ketamine hydrochloride (Vetalar™, 230 mg/kg) and positioned in a stereotaxic frame (Kopf Instruments, Tujunga, CA, USA). Animals assigned to the lesion group received a unilateral injection of lactacystin (10 µg in 2.5 µl; L6785, Sigma-Aldrich, Poole, UK) into the L-MFB (AP: −4.4 mm, ML: −1.5 mm lateral from bregma and −7.8 mm ventral to dura) [31]. Lactacystin was dissolved in 0.9% saline (pH 7.4) immediately prior to use and stored on ice to prevent degradation. Injections were performed at a rate of 1 µl/min using a motorized syringe pump and the needle was slowly withdrawn 5 min after lesioning to minimize diffusion of toxin into the injection tract. Anesthesia was reversed 1 hr after induction by subcutaneous (s.c.) injection of atipamezole hydrochloride (Antisedan®, 5 mg/kg). Animals assigned to the control group underwent identical surgery, but received an injection of 0.9% saline. Post-operative care included analgesia (buphrenorphine, 0.3 mg/kg s.c. during the first 48 hr), fluid-replacement (4 ml glucosaline solution i.p.) and mashed high-nutrient food pellets during the first week after surgery. Animals were weighed and semi-quantitatively scored daily for neurological deficits using a general neurological rating scale [32]. This was done daily in a blinded fashion until the end of the experiment.

### Behavioural testing

Prior to testing (training or trial) animals were acclimatized to the testing room for 30 min.

#### Grip strength meter test

Forelimb motor dysfunction was assessed using a grip strength meter (GSM; TSE Systems, Bad Homburg, GER) as previously described [11], [33].

#### Accelerating rotarod

Performance on the accelerating rotarod test (TSE systems, Bad Homburg, GER) was assessed as previously described, with modification [34]. Briefly, animals were assessed pre-operatively at five speeds of rotation: 8, 10, 12, 14 and 16 rpm to establish a baseline performance. Animals were allowed to remain stationary at 0 rpm for 10 sec, after which the speed was increased to 8 rpm for 10 sec and then progressively to 10, 12 and 14 rpm for 10 sec each. The highest speed, 16 rpm, was maintained for 200 sec until the 4 min test period elapsed. These rotational speeds were chosen so that sham (uninjured) animals would not fall off during the test. A rat was considered failing the 4-min period test if (a) it fell off the device before the time period elapsed or (b) it simply gripped the rungs and spun for two consecutive revolutions rather than actively walking on the rotating rods. Rats from both groups were tested twice daily (once each morning and afternoon) over a 3-day period at 1, 3 and 5 wks post-surgery. The means of the test results were used for statistical analysis.

#### Rotational asymmetry

Apomorphine-induced rotation (0.1mg/kg) as an index of nigrostriatal damage was evaluated in a bank of eight rotameter bowls as previously described [11], [35]. Lesioned animals that did not reach a cut-off value of >5 contralateral turns, per minute, (indicative of ∼70% nigral cell loss) were excluded from further analysis.

### Magnetic resonance imaging

*In vivo* T_2_-weighted (T2W) MR images were acquired using a 7.0 T horizontal small bore magnet (Varian, Palo Alto, CA, USA) with a custom built head RF coil (David Herlihy, Imperial College London) linked to a LINUX-based control console running VnmrJ acquisition software (v2.3, Varian, Palo Alto CA, USA), using a multi-echo, multi-slice spin-echo pulse sequence (MEMS), with the following scan parameters: FOV = 35 mm×35 mm; matrix = 192×192; *TR* = 4200 ms; *TE* = 10, 20, 30, 40, 50, 60, 70, 80 ms; 4 averages, total scan duration 54 minutes, as previously described [11]. Fifty contiguous 500 µm-thick coronal slices were acquired ensuring complete coverage of the brain. Post-acquisition, MR images were visually inspected for motion or intensity artefacts and scans displaying such were excluded. On this basis, the final *N* numbers for MR image analysis at each time-point were lactacystin *N* = 7 and saline *N* = 5. All echo times (10–80 ms) were summed and converted to ANALYSE 7.5 (Mayo Clinic, Rochester, USA). Quantitative T_2_ relaxation maps were also obtained by a mono-exponential fit of the eight multi-echo images (TE = 10–80ms) using VnmrJ software [11].

### MR image analysis

#### Signal intensity measurements

Signal intensity (SI) values for T_2_ were measured in both the contralateral and ipsilateral hemispheres of saline and lactacystin-lesioned animals, in the corpus striatum (STR) and the substantia nigra (SN) from quantitative T_2_ maps as previously described [11].

#### Manual segmentation volumetric analysis

The volume of the whole brain (WBV), ventral midbrain (VM), corpus striatum (STR), cerebral cortex (CTX), hippocampus (HPC), cerebellum (CB) and lateral ventricles (LV), were measured using a manual segmentation approach [36], as described in our previous study [11]. Thickness of the primary motor cortex (M1) and the barrel field of the primary somatosensory cortex (S1BF) were also measured on *in vivo* MR images, as previously described [37], [38].

#### Deformation based morphometry

A single baseline scan from a control animal was chosen, based on scan-quality, as a canonical reference (CR), which defines an anatomical space for analysis. The orientation of the CR was further standardised (sCR) by rigidly registering the x-axis mirror (mCR) to the original CR and applying the halfway transformation. An approximate brain-region for registration was created on the sCR using the 3D region-growing tools available in MRIcroN (MRIcroN: http://www.sph.sc.edu/comd/rorden/MRicron/). The brain mask was dilated outside the brain boundary and foreground: background voxels were weighted 1000∶1. All scans were normalised to the sCR using FLIRT registration [39], [40] with 9 degrees of freedom (dof) and the –nosearch option followed by transformation into the sCR space. All scans were corrected for intensity inhomogeneity artefact using N3 [41]. High signal-to-noise mean images for each group (control, lesion) at each time-point (baseline, 1, 3 and 5 wks) were created by averaging the relevant registered intensity-corrected images. High-dimensional fluid-registration [42] was used to warp each lesion-mean to the normal-mean at the corresponding time-point; this approach removes growth-related confounds by comparing each time-point against an age-matched control. Maps of localised inter-group volume differences were computed from the Jacobian determinant of the non-rigid transformation at each time-point. Relative differences in global volumetric scaling between groups computed from the 9-dof registration were combined with Jacobian results to produce maps of total average volume-difference between groups.

### Tissue collection

Animals were terminally anaesthetised by a sodium pentobarbital overdose (60 mg/kg i.p.) and transcardially perfused with 0.9% saline, followed by ice-cold 4% paraformaldehyde (PFA) in 0.2 M phosphate buffer, pH 7.4. Brains were rapidly dissected out, post-fixed for 24 hours and cryo-protected in buffered 30% sucrose. Serial coronal sections (40 µm) were cut on a freezing microtome at −20°C and stored in cryoprotective solution containing 0.05% sodium azide at −20°C until processed for immunohistochemistry.

### Immunohistochemistry

Tissue sections processed for stereology were immunostained free floating using a standard immunoperoxidase method as described previously [11]. To identify dopaminergic neurons in the SNc and fibres in the striatum, sections were stained with rabbit anti-tyrosine hydroxylase (TH, AB151, Chemicon, 1∶3000). To examine global neuronal loss, tissue sections were stained with mouse anti-Neuron specific nuclear protein (NeuN, MAB377, Chemicon, 1∶1000).

To visualize α-synuclein aggregation in dopaminergic neurons, tissue sections were processed for fluorescence microscopy. Sections were rinsed 3×10 min with PBS, followed by 1 hr incubation in blocking solution (10% normal goat serum, 0.3% Triton X-100 in PBS) at room temperature (RT). Sections were double-labelled with mouse anti-α-synuclein (610786, BD Biosciences, 1∶100) and rabbit anti-tyrosine hydroxylase (TH, AB151, Chemicon, 1∶3000). All primary and secondary antibodies were diluted in blocking solution. All incubations with primary antibodies were overnight at 4°C, followed by incubation with appropriate secondary antibodies conjugated to a fluorescent moiety for 2 hr at RT (ALEXA488 1∶500, ALEXA555 1∶1000; all from Molecular Probes, Invitrogen, UK). Tissue sections were washed thoroughly in PBS and mounted in vectashield containing 4′, 6-diamidino-2-phenylindole (DAPI) (Vector Laboratories, UK) on glass slides. Antibody specificity was confirmed in adjacent tissue sections with the primary or secondary antibody omitted.

### Post-mortem histological analysis

#### Quantification of neuronal loss in the SNc and striatum using the optical fractionator probe

Unbiased estimates of the number of TH-positive (TH+) neurons within the SNc in saline and lactacystin-lesioned animals were obtained as previously described [11]. To obtain unbiased estimates of the total number of neurons in the striatum in each group, the optical fractionator probe was utilized. The number of NeuN-positive cells were counted in every 12^th^ serial section of the striatum with the reference volume defined from +2.2 mm to −1.4 mm from bregma [43]. In each section, the area of analysis was traced at ×2.5 magnification and Cavalieri's method was used to estimate the volume of the reference region [44], [45]. These values were compared to MRI-derived measurements of striatal volume to check the reproducibility of these *in vivo* measurements. The number of neurons was sampled in each tissue section using counting frames (80×80 µm), systematically distributed with known x and y steps throughout the region from a random starting point. At least 20–25 counting frames were sampled per section, under ×60 magnification. Cells were only counted if they did not touch the exclusion lines and if they came into focus within the 18-µm thick optical dissector, with 2-µm guard zones. Estimates of neuronal number were generated by Stereo Investigator software. The coefficients of error (CE) were calculated according to the procedure of West and colleagues with values <0.10 accepted [46]. To ensure the absence of bias in cell counting, slides were coded such that the operator was blinded to the surgical status of the animal.

#### Post-mortem cortical thickness measurements

To replicate MRI measurements post-mortem, the thickness of the M1 and S1BF were measured using the contour measurement tool in Stereo Investigator software using the same microscope set-up described for stereology analysis. M1 thickness was measured 2 mm lateral from midline, on every 12^th^ serial section, at slices corresponding approximately to +3.00 mm, +2.52 mm and +2.04 mm from the cingulum to the brain surface. S1BF thickness was measured 6 mm from midline on every 12^th^ serial section corresponding approximately to Bregma −0.36 mm, −0.84 mm and −1.32 mm from the external capsule to the brain surface. The mean thickness of M1 and S1BF cortices was calculated for each subject from the average of ten lines drawn per structure. The mean values for each subject, in each hemisphere were then averaged to give an overall group mean.

#### Optical density measurements

Optical density of TH+ fibres in the corpus striatum was measured as previously described with modification [11], [47].

### Statistical analysis

All data are expressed as the mean ± SEM. Behavioural data, MRI volumes and T_2_ SI measurements were analysed using repeated measures (RM) two-way analysis of variance (ANOVA) with lesion and time as main effects. *Post-hoc* tests were performed using Bonferroni's correction for multiple comparisons. Post-mortem data were analysed using two-tailed students t-test. Correlations between regional brain volume changes, histology and motor behaviour across time were evaluated for all subjects (both saline controls and lactacystin-lesioned animals) using linear multiple regression models (variables entered) or a Pearson's correlation, as appropriate. All statistical analysis was performed in SPSS (v17.0; SPSS Inc. Woking; UK).

## Results

### Lactacystin microinjecton induces motor behaviour deficits

Lesioned animals, but not saline controls, consistently showed deficits in spontaneous motility, possibly reflecting developing bradykinesia in these animals. A clear ipsiversive deviated posture with spontaneous circling and dystonic deviation of the body axis towards the ipsilateral site of injection was also observed in lesioned animals, but not saline-controls. This was reflected in an overall increase in median neurological score, up to day 14, after which neurological dysfunction remained unchanged (Figure S1).

**Figure 2:**
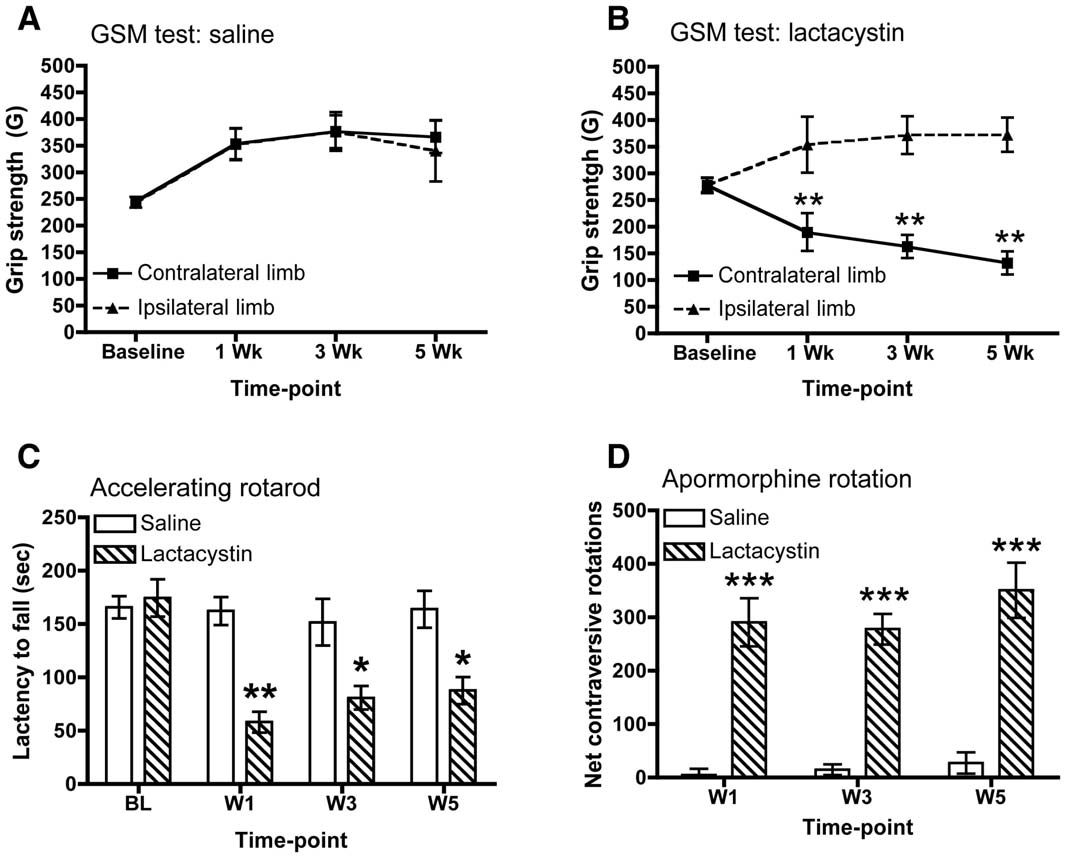
Parkinsonian-like motor phenotype in Lactacystin-lesioned animals. (A) Saline-injected controls show no deficits in the GSM test for either limb at any time-point. (B) Lesioned animals develop a progressive impairment in the grip strength of the contralateral forelimb in the GSM test. Data shown in (A, B) are mean grip force (G) ± SEM. **p<0.01 contralateral limb vs. ipsilateral limb. (C) Lesioned animals, but not saline controls show motor co-ordination deficits as evidenced by shortened latency to fall in the accelerating rotarod test at all time-points post-surgery. Data shown are mean latency to fall (sec) ± SEM. *p<0.05; **p<0.01; saline vs. lactacystin-injected. (D) Lactacystin-lesioning induces significant contralateral circling in response to apomorphine challenge (0.1 mg/kg s.c.) at all time-points post-surgery. Data shown are mean net contraversive rotations ± SEM. ***p<0.001; saline (*N* = 5) vs. lactacystin-injected (*N* = 7).

Forepaw grip strength improved with time in saline controls [*F*_(3,30)_ = 13.49, *p*<0.0001, Figure 2A]. By contrast, in lesioned animals [*F*_(1,36)_ = 19.95, *p*<0.001, Figure 2B], there was a significant impairment in grip strength of the contralateral forepaw by wk 1 (*p*<0.05), which was maintained at wk 3 (*p*<0.01) and wk 5 (*p*<0.01). As there was no significant difference in forepaw grip strength prior to the administration of lactacystin (i.e. baseline), grip strength in both forepaws evolved differently over time (*F*_(3,36)_ = 11.79, *p*<0.001). Forepaw grip strength was therefore significantly affected by administration of lactacystin into the L-MFB by week 1, but did not exhibit a progressive worsening of this deficit.

Performance on the accelerating rotarod revealed a significant impairment of lesioned animals (*F*_1,36_ = 17.48; *p*<0.0001, Figure 2C). However, this deficit was only evident after baseline (*F*_(3,36)_ = 7.769, *p*<0.01), although both groups' performance changed with time (*F*_3,36_ = 9.79; *p*<0.0001). Lesioned animals showed a significantly shorter latency to fall compared to saline-controls at wk 1 (*p*<0.001), wk 3 (*p*<0.01) and wk 5 (*p*<0.01). By wk 3 and 5, lesioned animals performance appeared to slightly recover, although it remained significantly impaired compared to saline controls throughout. Thus, motor co-ordination impairment was greatest at wk 1.

To probe striatal dopamine availability, animals were challenged with the dopamine D_2_ receptor agonist apomorphine. Lesioned animals exhibited a significant increase in contraversive rotations (*F*_1,26_ = 159.0; *p*<0.0001) at all time-points tested (*p*<0.01; Figure 2D). On average, lesioned animals displayed net contraversive turns of 6–7 turns/min, with locomotor activity increasing steadily after 5 min of drug administration and maintained for approximately 35 minutes, at which point locomotor activity decreased back to baseline. One lesioned animal did not demonstrate significant (>5 turns/min) contralateral circling and was thus excluded from all further analysis. Additional lesioned animals, but not saline controls, sacrificed at wk 1 and wk 3 showed identical rotational behaviour (*data not shown*).

### Substantia nigra signal intensity evolves longitudinally following microinjection of lactacystin

**Figure 3:**
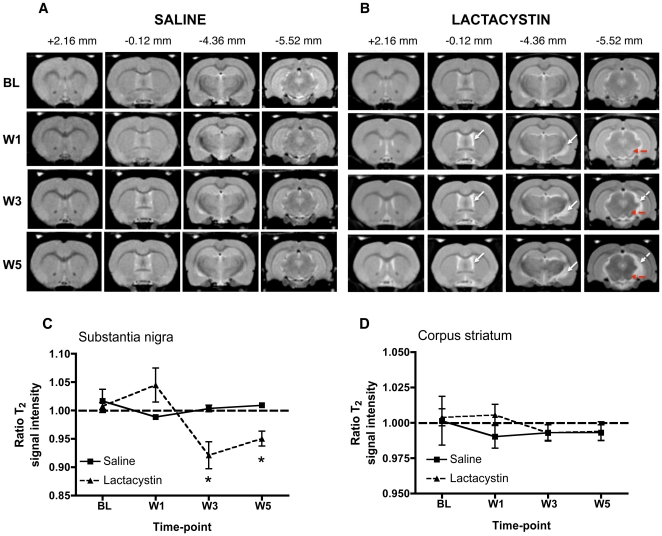
Representative longitudinal *in vivo* T2W MR images acquired from (A) saline-injected controls and (B) lactacystin-lesioned animals. MR images are shown at four coronal levels (approximate distances from bregma are shown in mm). Note the absence of visible pathological changes in saline injected controls. By contrast, in lesioned animals hypertrophy of the lateral ventricles and deformation of the ventral midbrain with concomitant increase in CSF space (dashed arrows in B) may be observed post-lesion. Apparent contrast changes were also present in the area of the substantia nigra (solid arrows in B) with time. (C) No significant alteration in T_2_ signal intensity was observed at any time-point between groups in the striatum. (D) By contrast, a clear temporal evolution of changes in T_2_ signal intensity are apparent in the substantia nigra of lactacystin-lesioned animals compared to saline controls, ranging from hyperintense signal at wk 1 to hypointense signal at wk 3 and wk 5. Data shown are the mean ratio of T_2_ signal intensity between the contralateral non-injected and ipsilateral injected brain hemispheres in each group ± SEM. *p<0.05; saline (*N* = 5) vs. lactacystin (*N* = 7).

To probe the underlying pathology responsible for inducing this behavioural phenotype, longitudinal T_2_-weighted MR images were acquired to measure the evolution of structural deformations, as well as changes in signal intensity that indicate a change in tissue composition. Remarkable changes in the contrast of the MR images were observed in the ventral midbrain across time in lesioned animals, which were absent in saline controls (Figure 3A, B). A hyperintense signal in an area approximate to the SN was present at wk 1 post-lesion, which evolved into an area of hypointense signal at later time-points. Quantification revealed longitudinal changes in the ratio of T_2_ SI between the ipsilateral and contralateral hemispheres, in the SN (*F*_(1,30)_ = 5.87; *p*<0.05, Figure 3C). This SI evolution in lactacystin animals was only present post-lesion (*F*_(1,30)_ = 7.39; *p*<0.01), but there was no overall effect of time (*F*_(1,30)_ = 5.36; *p*>0.05) indicating that the signal change was not associated with normal aging. The hyperintense contrast in the SN of lesioned animals at wk 1 post-lesion, however, failed to reach statistical significance. By contrast, at later time-points, the T_2_ SI ratio was significantly reduced (hypointense contrast) in the SN of lesioned animals at wk 3 (*p*<0.05) and wk 5 (*p*<0.05). No significant alterations in T_2_ SI ratio were observed in the STR at any-time point, in either lesioned animals or saline controls (Figure 3D). Specific changes in T_2_ SI are therefore only observed in the SN in this model, suggesting that these reflect particular aspects of pathology that are changing the signal characteristics of this region.

### Microinjection of lactacystin produces a progressive pattern of brain structural changes

Visual inspection of MR images revealed a progressive enlargement of the lateral ventricles (LV), accompanied by conspicuous atrophy in the ventral midbrain (VM) and ventral thalamus, with a concomitant increase in cerebrospinal fluid (CSF) space in the lesioned group that was absent in saline controls (Figure 3A, B). Longitudinal changes in brain volume for major brain structures in saline and lesioned animals were investigated utilising a manual segmentation analysis of *in vivo* MR images (see Table S1 for summary of statistical analyses).

**Figure 4:**
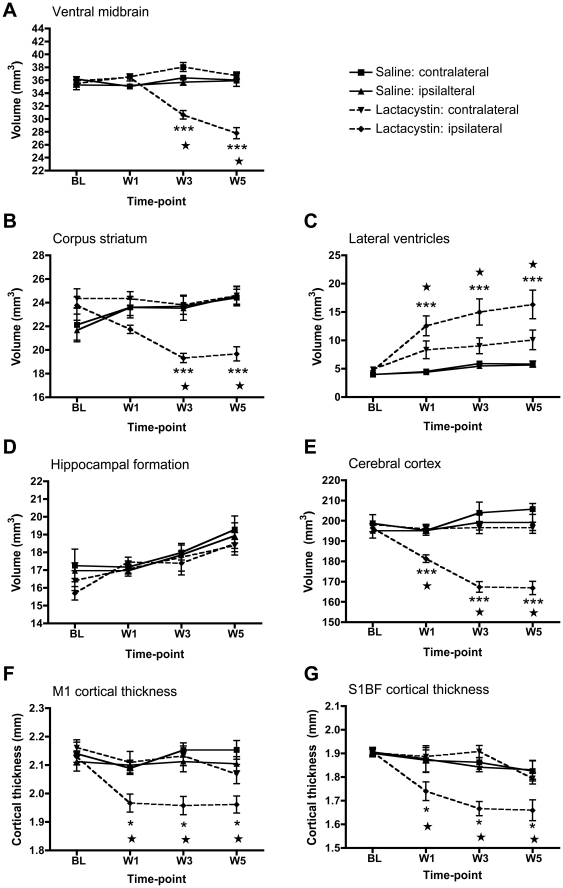
Time-course of regional brain volumetric changes in saline and lactacystin-injected animals. Significant tissue volume change was observed in (A) the ipsilateral ventral midbrain and (B) ipsilateral corpus striatum at 3 and 5 wks post-surgery compared to the non-injected contralateral hemisphere and both brain hemispheres in saline controls. (C) Lateral ventricle hypertrophy and (D) cortical atrophy were present from 1-wk post-surgery and maintained at 3 and 5 wks in lesioned animals, but not saline controls. (E) No significant change in the volume of the hippocampus was observed in either hemisphere in either group at any time-point. Significant thinning of the primary motor (F) and primary somatosensory cortex were also present from wk 1 post-lesion in lactacystin-injected animals, but not saline controls. Data shown are mean volume (mm^3^ A–E) or thickness (mm, F, G) ± SEM. ***p<0.001 ipsilateral hemisphere vs. non-injected contralateral hemisphere in lactacystin-injected animals; ★★★p<0.001 ipsilateral hemisphere of lactacystin-lesioned animals vs. ipsilateral hemisphere of saline controls. Saline, *N* = 5, lactacystin, *N* = 7.

Lactacystin microinjection into the L-MFB induced significant gross morphological changes that affected whole brain volume (WBV) compared to saline controls at wk 3 (*p*<0.05) and wk 5 (*p*<0.05), but not wk 1 (Figure S2A). These changes were contained within the cerebrum as no significant change in the volume of the cerebellum over time was observed (Figure S2B). For sub-cortical brain regions, a significant volume decrease in the ipsilateral ventral midbrain (VM) appeared from wk 3 onwards, increasing by wk 5, suggesting a progressive degeneration of this structure in lesioned animals. A significant volume decrease was observed in the ipsilateral STR from Wk 3 onwards in lesioned animals compared to saline controls (Figure 4A, B). A trend towards a decreased ipsilateral STR volume was observed at wk 1, but this failed to reach statistical significance (Figure 4B).

Volume changes were not confined to the basal ganglia following lactacystin microinjection. Significant hypertrophy of the ipsilateral LV was observed in lesioned animals at all post-lesion time points (*p*<0.01; Figure 4C). A trend towards hypertrophy of the contralateral LV of lesioned animals was also observed, but this failed to reach statistical significance (Figure 4C). Additionally, a significant reduction in the volume of the ipsilateral cerebral cortex (CTX) was observed at wk 1 (*p*<0.05) in lesioned animals compared to saline controls. This increased further at wk 3 (*p*<0.01) and wk 5 (*p*<0.01; Figure 4E). Within the CTX, lesioned animals had significantly thinner M1 and S1BF compared to saline controls at wk 1 (*p*<0.05), wk 3 (*p*<0.01) and wk 5 (*p*<0.01, Figure 4F, G). No significant changes were observed in the thickness of either the M1 or S1BF in the contralateral hemisphere in either treatment group (Figure 4F, G). However, no significant volume change was observed in the hippocampal formation in either hemisphere in either saline or lesioned animals (Figure 4D).

To examine the relationship between unspecific whole brain volume decreases and specific regional volume changes, MRI volume measurements (saline and lactacystin) for the ipsilateral hemisphere were correlated using Pearson product moment analysis (Table 1). This revealed a highly significant positive correlation between ipsilateral CTX volume alone and WBV at wk 1. By wk 3, both the ipsilateral VM and ipsilateral STR volume in addition to ipsilateral CTX volume were associated with changes in WBV. Notably, decreases in WBV only reached statistical significance from wk 3, indicating that tissue volume decrease in the CTX alone at wk 1 is insufficient to induce a change in WBV in lactacystin-lesioned animals, but that changes in multiple brain structures are required. By contrast, ipsilateral LV volume was negatively correlated with WBV, indicating that as WBV shrinks, ventricular volume increases. At wk 5, only ipsilateral VM volume was significantly correlated to WBV, implying that a progressive degeneration of the ipsilateral VM is primarily influencing changes in WBV at this stage of degeneration, but no other region.

**Table 1:** Correlations between *in vivo* whole brain volume and regional brain volume changes across time.

Brain region	WBV(wk 1)	WBV(wk 3)	WBV(wk 5)
Ventral midbrain	.*196*	.815**	.*688* *
Corpus striatum	.*412*	.786**	.*538*
Cerebral cortex	.712**	.*680* *	.*525*
Lateral ventricles	*−.322*	*−.593* *	*−.287*
M1 cortex	.*530*	.*495*	.*273*
S1BF cortex	.*431*	.*384*	.*012*

*Correlation is significant at the 0.05 level, **correlation is significant at the 0.01 level. *(Abbreviations: WBV, whole brain volume).*

To examine whether these MRI changes are related to behavioural impairment in this model, equation models were derived using a multiple linear regression to predict final behavioural impairments based on MRI measurements (Table 2). Overall, thickness of the ipsilateral CTX was revealed as the best predictor of forepaw grip strength, whilst M1 cortex thickness best predicted performance on the accelerating rotarod. Unexpectedly, ipsilateral LV volume best predicted the number of contraversive rotations after apomorphine administration. Neither ipsilateral STR volume, nor ipsilateral VM volume, were significant predictors of behavioural outcome for any behavioural test performed.

**Table 2:** Multiple regression analysis between MRI and behavioural data.

Behavioural test	Equation model predicting behaviour at wk 5
GSM test	= −921.63+(.914*CTX volume)
Accelerating rotarod	= −539.38+(.826*M1 cortex thickness)
Apomorphine rotation	= −56.66+(.846*LV volume)

*(Abbreviations: CTX, cortex; M1, primary motor cortex; LV, lateral ventricle).*

### Deformation based morphometry analysis of brain structure

**Figure 5:**
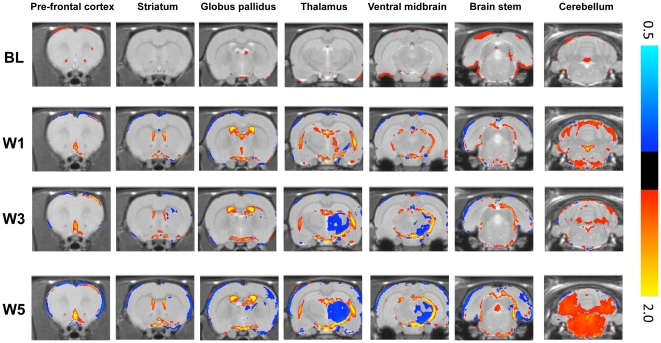
Deformation Based Morphometry confirms and extends manual segmentation analysis. Comparisons between the saline and lactacystin mean normalised MR images are shown for each time-point (*p<0.02 uncorrected*). The coloured overlay shows the scale factor of local apparent volume differences (on a logarithmic scale). Red/yellow indicates local tissue volume expansions, whilst blue/cyan indicates local tissue volume contractions.

This analysis identified several areas of local tissue volume change in cortical and sub-cortical areas, which evolved with time (Figure 5). At wk 1, increases in CSF space in lactacystin-treated animals are evident and persist until wk 5. At wk 3, local volume decreases in the ipsilateral VM were evident and continued to expand at wk 5. Similarly, volume decreases were observed in the ipsilateral STR at wk 3 and 5, but interestingly, DBM analysis suggests that volume change is confined to the lateral and medial dorsal regions of the ipsilateral STR. Decreases in the ipsilateral CTX volume were not readily apparent from wk 1 onwards, but small, localized areas of volume decrease may be observed in frontal cortical areas. By wk 3 and wk 5 however, cortical volume decreases are widespread in the ipsilateral hemisphere. Intriguingly, DBM analysis also suggests volume decreases in the contralateral CTX by wk 5. No local tissue volume changes were observed in the hippocampus. Apparent changes in the cerebellum were a reflection of the general brain volume decrease that resulted in a larger gap between cerebrum and cerebellum. Taken together, DBM analysis confirms the manual segmentation results.

Notably, DBM identified additional local tissue volume changes in areas of the brain that were immeasurable using the manual segmentation approach. For instance, clear volume decreases were observed in the ipsilateral ventral thalamus and globus pallidus from wk 3 onwards (Figure 5). Volume decreases were also observed in other ipsilateral cortical regions from wk 3 onwards, including areas approximate to the piriform and lateral entorhinal CTX (Figure 5). DBM detected additional significant expansion of tissue volume in an area approximate to the SN at wk 1, which was replaced by decreases at wk 3 and wk 5.

### *In vivo* MRI atrophy reflects post-mortem histopathological changes

**Figure 6:**
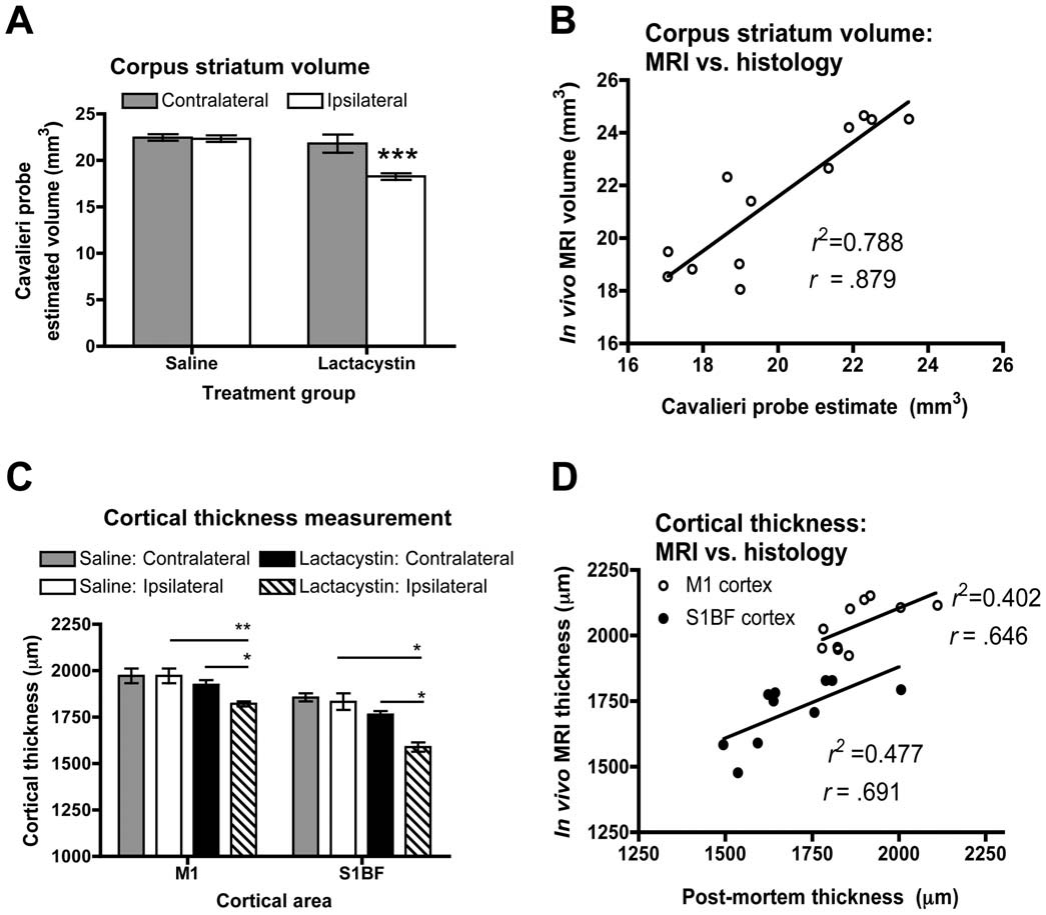
Post-mortem confirmation of *in vivo* MRI signal changes. (A) Cavalieri estimator probe measurement of corpus striatum volume post-mortem in saline and lactacystin-injected animals reveals a significant decrease in the volume of the ipsilateral striatum in lesioned animals, (***p<0.01). (B) Linear regression analysis reveals a strong correlation between measurement of striatal volume in both groups from either MRI or post-mortem histology (*r* = . 879). (C) Cortical thickness measurements post-mortem confirms cortical thinning in the M1 and S1BF cortices of lactacystin-lesioned animals but not saline controls, consistent with MRI data. (*p<0.05; **p<0.01). (D) Linear regression also reveals a robust correlation between cortical thickness measurements in the M1 and S1BF by MRI or from post-mortem histological sections (*r* = . 646 and .691; respectively). Data shown in (A) and (C) are mean volume or thickness, respectively, ± SEM. Saline, *N* = 5, lactacystin, *N* = 7.

To confirm *in vivo* MRI measurements, the volume of the STR was determined post-mortem by stereology using the Cavalieri probe estimator, which confirmed a significant decrease in the volume of the ipsilateral STR in lesioned animals, compared to saline controls (*p*<0.0001; Figure 6A). No significant change was observed in the contralateral STR in either group (Figure 6A). Overall, there was excellent correspondence between *in vivo* MRI and post-mortem histological measurements of STR volume (*r* = .879; *p*<0.001; Figure 6B). Similarly, cortical thinning in the M1 and S1BF CTX measured from MR images was confirmed post-mortem in the M1 (p<0.01) and S1BF (p<0.01) (Figure 6C). No change was observed in the contralateral hemisphere in either group. Good correspondence was observed between *in vivo* MRI and post-mortem cortical thickness measurements for both M1 (*r* = .646; *p*<0.05) and S1BF (*r* = .691; *p*<0.05; Figure 6D).

### Neuropathology underlying tissue volume changes

**Figure 7:**
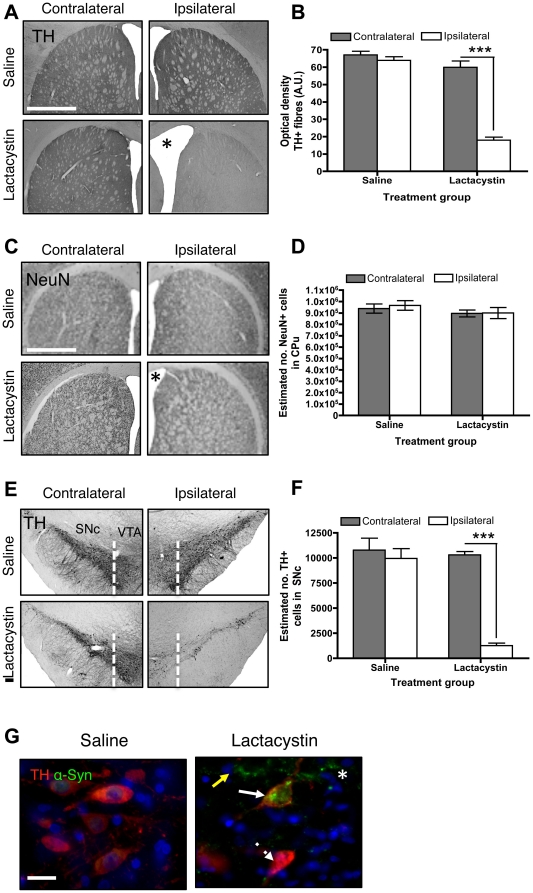
Neuropathological alterations following lactacystin lesioning of the nigrostriatal system. (A) Lactacystin microinjection induces substantial TH+ fibre loss in the ipsilateral striatum, accompanied by ventricular enlargement (*). (B) Quantification of TH^+^ fibre density in lesioned (*N* = 7) and control animals (*N* = 5). Data shown are mean TH fibre density (A.U) ± SEM; ***p<0.001; ipsilateral vs. contralateral hemisphere. (C) Striatal volume change in lesioned animals is not associated with gross neuronal loss confirmed by (D) optical fractionator counts of striatal neuron number. Data shown are the mean number NeuN+ cells ± SEM; ***p<0.001; ipsilateral vs. contralateral hemisphere. (E, F) Lactacystin microinjection induces significant loss of nigral TH+ cell bodies. Data shown are mean number TH+ cells in the SNc ± SEM. ***p<0.001; ipsilateral vs. contralateral hemisphere. (G) Loss of TH+ cells is accompanied by formation of α-synuclein immunopositive aggregates in lesioned animals compared to saline controls. Note the clear pattern and distribution of α-synuclein inclusions, with some surviving TH+ cells showing inclusion pathology (solid white arrows), some surviving TH+ cells without α-synuclein positive inclusions (dashed white arrows), TH-negative cells with α-synuclein positive inclusions (yellow solid arrows) and aggregates of α-synuclein in the brain parenchyma (asterisks). Images in (A, C, E) ×4 magnification, scale bar = 200µm, images in (G) ×40 magnification, scale bar = 20 µm.

To identify the specific neural substrates of structural changes, animals evaluated by serial MRI underwent post-mortem immunohistochemical evaluation of the integrity of the nigrostriatal system. At the level of the STR, lactacystin microinjection into the L-MFB resulted in a ∼70% reduction of TH+ fibres in the ipsilateral STR 5 wks post-surgery, accompanied by a visible hypertrophy of the LV, relative to the intact contralateral hemisphere. These changes were not observed in saline controls (*p*<0.001; Figure 7A, B). To establish whether volume change in the ipsilateral STR at Wk 5 reflects atrophy due to neuronal loss, the number of NeuN+ cells in the contralateral and ipsilateral striatum was quantified. This confirmed no significant difference in the number of NeuN+ cells in the ipsilateral striatum relative to the contralateral striatum in either saline or lactacystin-injected animals (Figure 7C, D). A significant increase in neuronal density (NeuN+ cells/mm^3^) in the ipsilateral striatum of lactacystin lesioned animals was observed (49,377±2771 vs. 41,260±1961 neurons/mm^3^; *p*<0.05), which was absent in saline controls (43,309±1925 vs. 41,804±1693 neurons/mm^3^; *p*>0.05). Taken together these data suggest striatal volume change in this model does not reflect neuronal loss *per se*.

At the level of the SNc, lactacystin microinjection into the L-MFB resulted in a substantial loss of TH+ cells in the ipsilateral SNc, but also the VTA, relative to the intact contralateral hemisphere at wk 5, which was not observed in saline controls (Figure 7E). Quantification by stereology confirmed a significant loss of TH+ cells (∼85%) in the SNc of lactacystin-lesioned animals relative to the intact contralateral hemisphere (*p*<0.0001; Figure 7F). No loss of TH+ cells was observed in either hemisphere of the SNc in saline controls (Figure 7F). Loss of nigral TH+ neurons was accompanied by a distinct pattern of α-syn aggregation in the SNc. Large irregular α-syn inclusions were observed in a low proportion of the rare surviving TH+ cells in the SNc at wk 5. Surviving TH+/α-syn- cells were also present, as were TH-negative cells that contained α-syn aggregates. Free aggregates of α-syn were also observed in the parenchyma. No α-synuclein (α-syn) aggregation was observed in saline-injected controls (Figure 7G).

To document the time-course of pathological changes in the nigrostriatal system, post-mortem immunohistochemical analysis was carried out in additional cohorts of lesioned and control animals, sacrificed at 1 and 3 wks post-surgery respectively. This revealed that in lesioned animals TH+ fibre loss in the ipsilateral STR, TH+ cell loss and the pattern of α-syn aggregation in the ipsilateral SNc was directly comparable at wk 1 and wk 3, to that observed at wk 5 (Figure S3).

### Lactacystin microinjection induces secondary neurodegeneration in the thalamus and ventral midbrain

**Figure 8:**
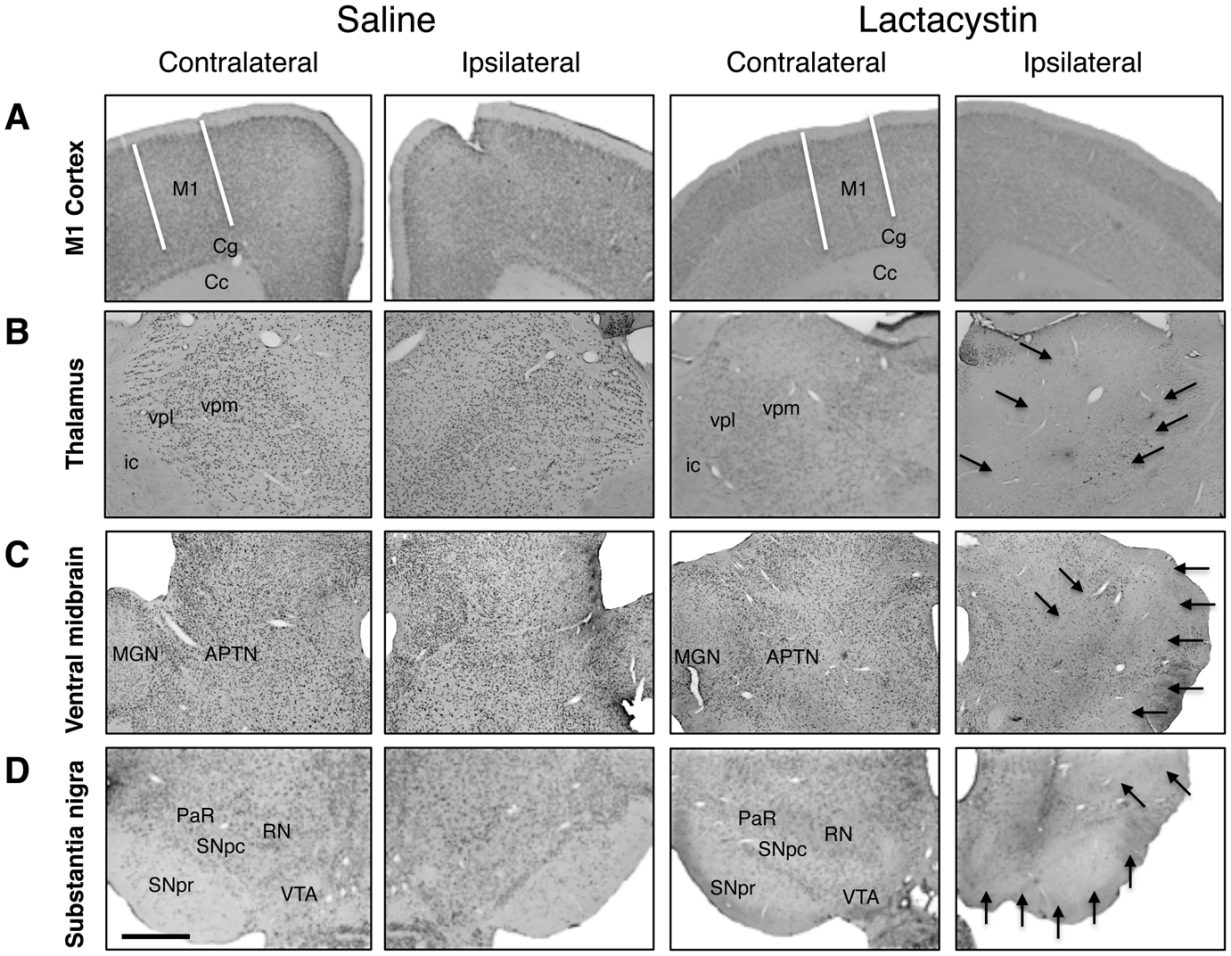
Qualitative analysis of neuronal loss in extra-nigral brain regions demonstrating MRI changes at week 5 post-lesion. (A) Lactacystin microinjection does not result in gross neuronal loss in the ipsilateral primary motor (M1) cortex. By contrast, compared to saline controls widespread neuronal loss is apparent (solid black arrows) in (B) ipsilateral ventral thalamic nuclei, (C) ipsilateral ventral midbrain extra-nigral nuclei and (D) substantia nigra pars compacta (SNc) in lesioned animals. Note the loss of neurons in the ventral tegmental area (VTA) and substantia nigra pars reticulata (SNr). All images ×4 magnification, scale bar = 200 µm. Abbreviations: M1, primary motor cortex; cg, cingulum; cc, corpus callosum; ic, internal capsule; vpl, ventral posterolateral thalamic nucleus; vpm, ventral posteromedial thalamic nucleus; mgn, medial geniculate nucleus; APTN, anterior pretectal nucleus; PaR, pararubral nucleus; RN, red nucleus. Saline, *N* = 5, lactacystin, *N* = 7.

To determine if volume changes in other brain regions are the result of secondary neurodegeneration following the primary insult caused by nigrostriatal destruction, the presence or absence of neuronal loss in the M1 cortex, thalamus, and ventral midbrain were assessed qualitatively from NeuN-stained tissue sections (Figure 8). No evidence of gross neuronal loss was observed in the M1 cortex (Figure 8A) or the S1BF (*data not shown*), in either saline controls or lesioned animals, at wk 5 (Figure 8A) nor at wk 1 (Figure S4) or wk 3 post-lesion (Figure S5). By contrast, in lesioned animals, but not saline controls, severe neuronal loss was observed in the ipsilateral ventral posterolateral (VPL) and ventral posteromedial (VPM) thalamic nuclei at wk 5 (Figure 8B). Similarly, in the ipsilateral ventral midbrain (VM) of lesioned animals, but not saline controls, extensive neuronal loss was observed in numerous nuclei including the red nucleus (RN), medial genticulate nucleus (MGN) and anterior pretectal nucleus (APTD) at wk 5 (Figure 8C). Extensive neuronal loss was also observed in the SNc at this time-point (Figure 8D). Additionally, the substantia nigra pars reticulata (SNpr) and the ventral tegmental area (VTA) were not free from the toxicity of lactacystin at wk 5 (Figure 8D). Analysis of lesioned animals sacrificed at wk 1 and wk 3 post-lesion revealed that neuronal loss in the thalamus and ventral midbrain was absent at wk 1 (Figure S4), but present at wk 3 post-lesion (Figure S5). These data are consistent with the evolution of substantial volume decreases identified by manual segmentation and DBM in the thalamus and ventral midbrain.

### Correlation analyses between brain volume change, behavioural dysfunction and post-mortem neuropathology

To identify whether MRI changes are associated with underlying nigral pathology, correlation analyses were performed between *in vivo* MRI measurements and post-mortem neuropathology measurements obtained at wk 5. Multiple regression analyses were also performed to identify which if any, *in vivo* MRI measurement best predicted nigrostriatal damage. Loss of both ipsilateral striatal TH+ fibres and nigral TH+ cells was significantly correlated with volume changes in the ipsilateral VM, STR, CTX, LV and M1 cortex thickness, but not S1BF cortex thickness or SI T_2_ measurements in the SN at this time-point (Table 3). Multiple regression analysis identified ipsilateral CTX volume as the best predictor of TH+ cell number at wk 5, whilst VM volume was the best predictor of TH+ fibre density at wk 5 (Table 4).

**Table 3:** Correlations between post-mortem measures of nigrostriatal damage and *in vivo* MRI measurements at wk 5.

	Histology	
MRI	TH+ cells SNc	TH+ fibres STR
VM volume	.*839* **	.*884* **
STR volume	.*749* **	.*835* **
CTX volume	.*840* **	.*882* **
LV volume	*−.676* *	*−.771* **
M1 thickness	.*751* **	.*841* **
S1BF thickness	.*494*	.*574*
SN T_2_ SI	.*576*	.*602*

*Correlation is significant at the 0.05 level,

**correlation is significant at the 0.01 level. (Abbreviations: VM, ventral midbrain; STR, corpus striatum; CTX, cortex; LV, lateral ventricle; M1, primary motor cortex, S1BF, primary somatosensory cortex barrel field; SN, substantia nigra; SI, signal intensity).

**Table 4:** Multiple regression analysis between MRI and post-mortem histology data.

Post-mortem histology	Equation model predicting histology at Wk 5
TH+ cells SNc	= −33198.18+(.829*CTX volume)
TH+ fibres STR	= −132.03+(.947*VM volume)

(*Abbreviations: CTX, cortex; VM, ventral midbrain; SNc, substantia nigra pars compacta; STR, corpus striatum*.)

Correlation analysis between nigrostriatal pathology and behavioural phenotype at wk 5 were also performed (Table 5). Highly significant correlations were found between loss of ipsilateral striatal TH+ fibres and nigral TH+ cells, as well as behavioural impairment in this model (Table 5). Multiple regression analysis confirmed that TH+ fibre density in the STR was the best predictor of behavioural impairment for all tests (Table 6). Multiple regression analyses determined which measurements, *in vivo* MRI or post-mortem histology, best predicted behavioural impairment in this model (Table 6). Interestingly, for both grip strength and apomorphine rotation, TH+ fibre density (post-mortem) was the strongest predictor over any MRI measurement. However, for performance in the accelerating rotarod, M1 cortical thickness as measured from MR images remained the best overall predictor of performance.

**Table 5:** Correlation analysis between post-mortem histology and behaviour.

	Behaviour		
Histology	GSM test	Accelerating rotarod	Apomorphine rotation
TH+ cells SNc	.*839* **	.*749* **	.*840* **
TH+ fibres CPu	.*884* **	.*835* **	.*882* **

*Correlation is significant at the 0.05 level,

**correlation is significant at the 0.01 level. Abbreviations: SNc, substantia nigra pars compacta; STR, corpus striatum.

**Table 6:** Multiple regression analysis between post-mortem histology and *in vivo* MRI with behavioural data.

	Equation models predicting behaviour		
Behaviour	*In vivo* MRI volumes	Post-mortem histology	MRI and Histology
Grip Strength	= −921.627+(0.914×CTX)	= 30.986+(0.930×Fibre Density)	= 24.759+(0.941×Fibre density)
Rotarod	= −539.377+(0.826×M1)	= 69.408+(0.694×Fibre Density)	= 539.377+(0.826×M1)
Rotameter	= −56.655+(0.846×LV)	= 477.563+(−0.926×Fibre Density)	= 586.086+(−0.955×Fibre Density)

(*Abbreviations: CTX, cortex; M1, primary motor cortex, LV, lateral ventricles.*)

## Discussion

Establishing the neurological basis of behavioural dysfunction is key to provide a better understanding of PD. To this end, using a combination of *in vivo* MRI and behavioural testing, we have identified the spatiotemporal sequence of tissue volume and signal intensity (SI) changes in animals lesioned with the proteasome inhibitor lactacystin, a rat pre-clinical model of PD, many of which have been reported from clinical neuroimaging studies of PD patients. Our manual analysis of brain volume changes is complemented by the application of deformation-based morphometry (DBM), which revealed additional pathology in lesioned animals, particularly in the thalamus, also reported in PD patients [48]. Using multiple regression analysis, we have established which *in vivo* MRI-based structural changes predict behavioural dysfunction. Consistent with our hypothesis, pathological changes in the basal ganglia, as well as extra-nigral structural alterations, particularly in the cortex, predict motor deficits in this model.

### Sequence of behavioural and neuropathological changes

Lactacystin lesioning resulted in significant motor impairments, consistent with previous reports in this model [11], [22], [23], [24], [25]. Notably, in all three tests, functional impairment in lesioned animals was maximal by wk 1 post-lesion. Interestingly, in the rotarod test, lesioned animals performance improved slightly by weeks 3 and 5 post-lesion. This may reflect learning of the test paradigm or may be related to intrinsic compensatory mechanisms. Post-mortem analyses of nigrostriatal integrity demonstrated that nigrostriatal destruction was already maximal by wk 1 in lesioned animals with no further increase at 3 or 5 wks. Overall, these data are consistent with an acute, rapid degeneration of nigral DA neurons and striatal DA deafferentation at this dose of lactacystin (10 µg), characteristic of the static non-progressive lesions generated following L-MFB administration of neurotoxins [49], [50].

As such, structural brain changes at wk 1 post-lesion may be the most relevant in this model. This time-point reflects end-stage PD, where nigrostriatal destruction is almost complete [51]. At wk 1, *in vivo* significant tissue volume changes were located only in the cortex, including thinning of the M1 and the S1BF cortex, which was confirmed post-mortem. Cortical thinning may therefore reflect the primary neuroanatomical alteration related to loss of nigral dopaminergic neurons in this model. Interestingly, these data are consistent with clinical MRI studies, reporting cortical volume changes in both early [52], [53], [54] and late-stage PD patients [55], [56], [57], although in later stages this is heavily influenced by co-morbidity with dementia [55], [58]. Cortical thinning is however, also present in non-demented late-stage PD patients [55], [58], [59], [60]. Importantly, however, imaging data from acute time-points post-lesion are required to confirm the timing of onset of cortical thinning in relation to nigral dopaminergic neuronal loss. Notably, we also did not detect any change in the volume of the hippocampal formation in lesioned animals, by manual segmentation. Several clinical imaging studies have identified decreased grey matter density in the hippocampus associated with depression and cognitive impairment in PD and Parkinson's disease dementia (PDD) patients [61], [62], [63]. This may be explained by the low sensitivity of the manual segmentation approach to detect subtle volume change. However, the more sensitive DBM method also did not detect hippocampal volume change. Thus, it may be that our model simply does not recapitulate the neuropathology associated with hippocampal volume change in PD. However, this study did have a small number of subjects, thus lowering statistical power to detect volume changes. Replication in a larger cohort with DBM analysis may identify hippocampal volume loss.

By contrast to wk 1, structural brain changes at wks 3 and 5 post-lesion are likely to reflect secondary neurodegeneration resulting from the initial insult in this model. Longitudinal MRI is therefore able to detect and chart the sequence of primary and secondary neuroanatomical changes caused by nigral neurodegeneration, which can be used to predict behavioural impairments.

### Predictive power of neuroanatomical changes detected by MRI

Linear multiple regression and correlation analyses reveal several important findings regarding the predictive power of brain volume changes in lesioned animals. Notably, decreased cortical volume was the best predictor of grip strength and rotarod performance, consistent with the notion that cortical changes are most likely linked to primary nigrostriatal degeneration. Interestingly, functional changes in cortical structures, including the anterior cingulate, motor and somatosensory cortex is associated with PD motor symptoms as evidenced by several functional MRI (fMRI)-based studies in the clinic [64], [65], [66], [67], [68] and in animal models [13], [14], [69], [70]. Unexpectedly, lateral ventricle (LV) volume best predicted the degree of apomorphine rotation. Although LV hypertrophy is reported in and correlates with clinical symptoms in PD patients [6], this is of little intrinsic value, since it is a measure of global, non-specific changes in brain structure. Overall, whilst these results do not imply an insignificant role of basal ganglia structures in PD-related motor behavioural impairment, they do suggest that subtle structural alterations in cortical regions may play a role in PD motor deficits.

However, in disagreement with clinical findings [3], [71], changes in nigral T_2_ SI are not linked to behavioural dysfunction in this model. However, this may be explained by the mismatching of rapidly developing neuronal death due to proteasome inhibition, versus the relatively delayed accumulation of iron in the SN in this model, which potentially underlies the hyperintense contrast in the SN [11], [23], [24], [25].

### Neurobiology underlying neuroanatomical changes

Whilst correlation analyses support the notion that cortical volume changes are related to motor dysfunction in this model, post-mortem validation of MRI signal changes is crucial before these can be accepted as surrogate markers. The excellent correspondence between *in vivo* MRI and post-mortem measurement of cortical thinning indicates that this measure may be a useful surrogate marker of motor impairment.

Establishing the mechanisms that underlie these changes remains however a challenge. Interestingly, cortical thinning in lesioned animals was not associated with gross neuronal loss at post-mortem. These data are consistent with the observation that cortical thinning may occur without neuronal loss in healthy elderly patients [72] and the lack of neuronal loss in the neocortex of cognitively impaired PD patients [73]. Notably, in this animal model STR volume changes *in vivo* were also not associated with neuronal loss post-mortem, consistent with findings in PD patients and other animal models [74], [75], [76]. Volume changes in the CTX and STR in this model may therefore be a consequence of the loss of DA innervation from the midbrain [47], [75], [76], [77]. Indeed, cortical DA depletion due to loss of mesocortical inputs results in dendritic spine remodeling [77] and altered synaptic morphology [78] in layer V cortical projection neurons, consistent with a functional re-organisation of these structures following loss of DA inputs from the midbrain. Potentially however, tissue volume changes in the CTX and STR may also reflect glial cell pathology. Indeed, whilst there are extensive studies on neurons, little attention has been paid to the effect of proteasome inhibition in glial cells, in particular, astrocytes. Notably, lactacystin has been reported to induce dose-dependent decreases in both proliferation and induces morphological changes in cultured cortical astrocytes [29], [30].

Post-mortem analysis revealed however, gross neuronal loss in the ventral midbrain and thalamus by 3 wks post-lesion, but not prior to this, consistent with the onset of volumetric change in these structures detected by MRI. Thus, volume change in these structures may reflect trans-synaptic neuronal loss, consistent with previous observations in lactacystin-lesioned animals [79]. However, a role for concomitant glial cell pathology cannot be excluded. Alternatively, thalamic neuronal loss may reflect a consequence of functional changes in basal ganglia circuitry due to DA depletion [80], [81], [82]. Consistent with this notion, thalamic neurodegeneration has been identified in post-mortem PD brain tissue [83], [84], [85], [86] and PD animal models [80], [87].

Crucially however, whilst dopaminergic neurons may be preferentially sensitive to proteasome inhibition [26] evidence suggests that synthetic proteasome inhibitors induce dose-dependent dopaminergic neuronal degeneration and are associated with a significant risk of non-specific neuronal and/or glial cell toxicity at higher doses [11], [22], [23], [27]. Thus, whilst the MRI changes observed in the current study are clearly linked to DA depletion, we cannot exclude the possibility that these may also reflect additional loss of other vulnerable neuronal or glial cell populations. A more progressive nigrostriatal degeneration, as reported with lower doses of lactacystin [23], may therefore provide more clinically-relevant correlations between behavioural deficits and MRI changes. Additionally, it is not clear if tissue volume and pathological changes in extra nigral regions are accompanied by pathological α-synuclein deposition in this model. Consequently, future studies examining both the dose-dependence of lactacystin-induced brain volumetric changes and further detailed post-mortem histological studies are required to fully understand the neuropathological correlates of brain volume changes in this model.

### Conclusions

Manual and voxel-based MRI analysis methods revealed that microinjection of lactacystin into the L-MFB results in a distinct sequale of structural alterations in the brain, many of which are consistent with descriptions of structural alterations in PD patients. Of these, cortical thinning exhibited a greater predictive power for the degree of functional impairment compared to all other *in vivo* measures. Structural changes in other brain regions may reflect secondary neuropathology due to non-specific toxicity of lactacystin or are the consequence of a functional reorganisation of basal ganglia circuitry following DA depletion. Consequently, these changes do not predict motor impairment *in vivo*. However, confirmation of these finding in a larger cohort of animals, at lower doses of lactacystin and in other pre-clinical models of PD, such as the rat 6-hydroxydopamine (6-OHDA) or 1-methyl-4-phenyl-1,2,3,6-tetrahydropyridine (MPTP) murine and non-human primate models will be important and significant advances. Nonetheless, these data highlight the importance of a comprehensive anatomical assessment beyond the primary insult in the nigrostriatal system. Furthermore, these data demonstrate that integration of MRI data with post-mortem neuropathology is not only essential to confirm the relevance of *in vivo* MRI findings to behavioural dysfunction. Moreover, this integrative approach provides a powerful model system with which to investigate the neuropathological correlates of structural MRI changes as observed in the brains of PD patients.

## Supporting Information

Figure S1 **Neurological scoring of animal health reveals lactacystin-lesioned (** ***N*** **= 7) animals develop a progressive increase in neurological score, consistent with subtle motor deficits and behavioural abnormalities.** Neurological scores increase to day 14 post-lesion and then become static. Saline controls (*N* = 5) display no gross neurological abnormalities.
(TIF)

Figure S2 **Longitudinal** ***in vivo*** **MRI detects a reduction in whole brain, but not cerebellum volume in lactacystin-lesioned animals (** ***N*** **= 7) compared to saline controls (** ***N*** **= 5).** Data shown are mean volume ± standard error. *p<0.05; saline vs. lactacystin.
(TIF)

Figure S3 **Time-course of nigrostriatal pathology induced by lactacystin microinjection into the L-MFB.** (A) Lactacystin microinjection induces substantial TH+ fibre loss in the ipsilateral striatum, accompanied by ventricular enlargement. (B) Quantification of TH^+^ fibre density in lesioned (*N* = 5) and control animals (*N* = 5) reveals this is maximal by week 1 post-lesion and does not progress further. Data shown are mean TH fibre density (A.U) ± SEM; ***p<0.001. (C, D) Quantification of nigral TH+ cell bodies in lesioned and control animals reveals this is maximal by week 1 post-lesion and does not progress further. Data shown are mean number TH+ cells in the SNc ± standard error ***p<0.001. (E) Loss of TH+ cells is accompanied by formation of α-synuclein immunopositive aggregates in lesioned animals compared to saline controls at week 1 and 3. Note the clear pattern and distribution of α-synuclein inclusions, with some surviving TH+ cells showing inclusion pathology (solid white arrows), some surviving TH+ cells without α-synuclein positive inclusions (dashed white arrows), TH-negative cells with α-synuclein positive inclusions (yellow solid arrows) and aggregates of α-synuclein in the brain parenchyma (asterisks). Images in (A, C, E) ×4 magnification, scale bar = 200µm, images in (G) ×40 magnification, scale bar = 20 µm.
(TIF)

Figure S4 **Qualitative analysis of neuronal loss in extra-nigral brain regions demonstrating MRI changes at week 1 post-lesion.** (A) Lactacystin microinjection (*N* = 5) does not result in apparent neuronal loss in the ipsilateral primary motor (M1) cortex at this time-point. Similarly, compared to saline controls (*N* = 5) no neuronal loss is evident in (B) ipsilateral ventral thalamic nuclei, (C) ipsilateral ventral midbrain extra-nigral nuclei. By contrast, substantial neuronal loss is present in (D) the substantia nigra pars compacta (SNc). Note also the loss of neurons in the nearby ventral tegmental area (VTA) and substantia nigra pars reticulata (SNr). All images ×4 magnification, scale bar = 200 µm. Abbreviations: M1, primary motor cortex; cg, cingulum; cc, corpus callosum; ic, internal capsule; vpl, ventral posterolateral thalamic nucleus; vpm, ventral posteromedial thalamic nucleus; mgn, medial geniculate nucleus; APTN, anterior pretectal nucleus; PaR, pararubral nucleus; RN, red nucleus.
(TIF)

Figure S5 **Qualitative analysis of neuronal loss in extra-nigral brain regions demonstrating MRI changes at week 3 post-lesion.** (A) Lactacystin microinjection (*N* = 5) does not result in apparent neuronal loss in the ipsilateral primary motor (M1) cortex at this time-point. By contrast, compared to saline controls (*N* = 5) widespread neuronal loss is already apparent (solid black arrows) in (B) ipsilateral ventral thalamic nuclei, (C) ipsilateral ventral midbrain extra-nigral nuclei and (D) substantia nigra pars compacta (SNc). Note also the loss of neurons in the nearby ventral tegmental area (VTA) and substantia nigra pars reticulata (SNr). All images ×4 magnification, scale bar = 200 µm. Abbreviations: M1, primary motor cortex; cg, cingulum; cc, corpus callosum; ic, internal capsule; vpl, ventral posterolateral thalamic nucleus; vpm, ventral posteromedial thalamic nucleus; mgn, medial geniculate nucleus; APTN, anterior pretectal nucleus; PaR, pararubral nucleus; RN, red nucleus.
(TIF)

Table S1 Summary of overall statistical results from repeated measures 2-way ANOVA for *in vivo* serial MRI measurements.
(DOC)
