# Supplementary figures and images for: Evolution of Extra-Nigral Damage Predicts Behavioural Deficits in a Rat Proteasome Inhibitor Model of Parkinson's Disease
Source: PLoS One. 2011 Feb 25;6(2):e17269. doi: 10.1371/journal.pone.0017269 (PMC3045435; doi:10.1371/journal.pone.0017269)

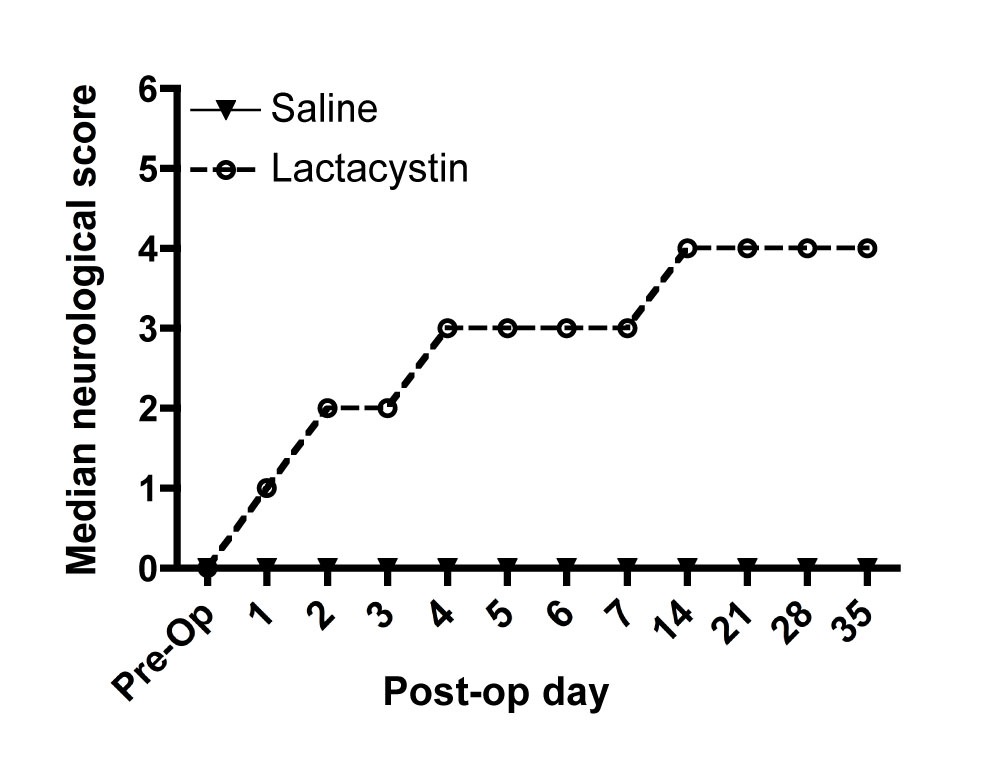

Supplement: Figure S1 — Neurological scoring of animal health reveals lactacystin-lesioned ( N = 7) animals develop a progressive increase in neurological score, consistent with subtle motor deficits and behavioural abnormalities. Neurological scores increase to day 14 post-lesion and then become static. Saline controls (N = 5) display no gross neurological abnormalities. (TIF) [file pone.0017269.s001.tif]

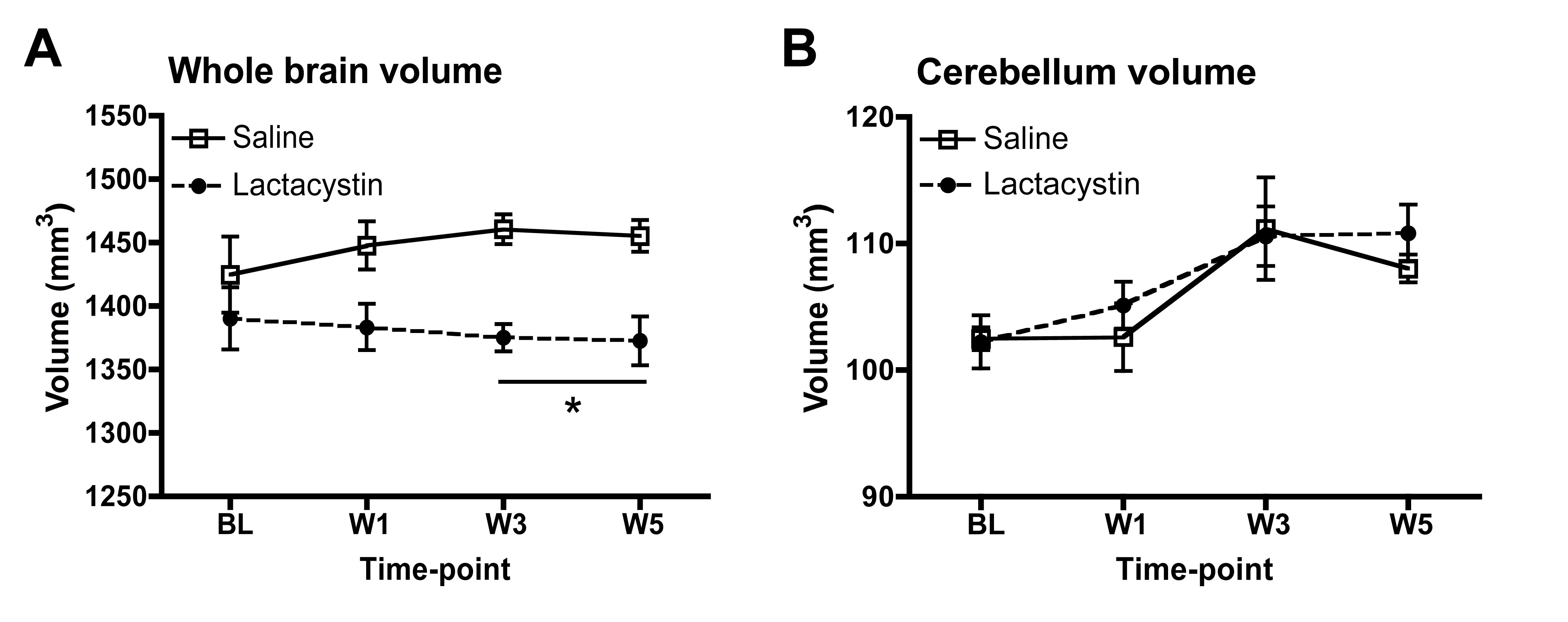

Supplement: Figure S2 — Longitudinal in vivo MRI detects a reduction in whole brain, but not cerebellum volume in lactacystin-lesioned animals ( N = 7) compared to saline controls ( N = 5). Data shown are mean volume ± standard error. *p<0.05; saline vs. lactacystin. (TIF) [file pone.0017269.s002.tif]

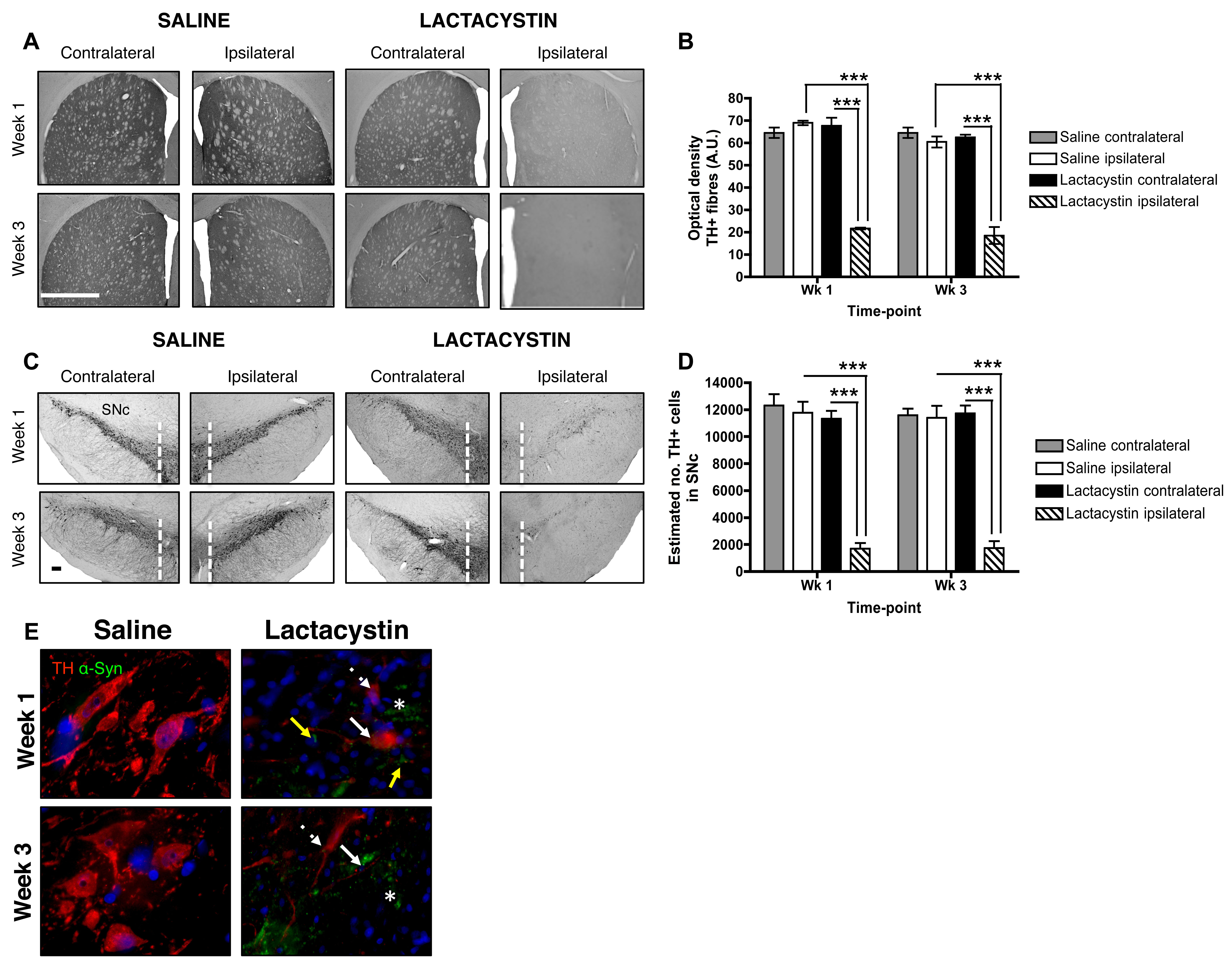

Supplement: Figure S3 — Time-course of nigrostriatal pathology induced by lactacystin microinjection into the L-MFB. (A) Lactacystin microinjection induces substantial TH+ fibre loss in the ipsilateral striatum, accompanied by ventricular enlargement. (B) Quantification of TH+ fibre density in lesioned (N = 5) and control animals (N = 5) reveals this is maximal by week 1 post-lesion and does not progress further. Data shown are mean TH fibre density (A.U) ± SEM; ***p<0.001. (C, D) Quantification of nigral TH+ cell bodies in lesioned and control animals reveals this is maximal by week 1 post-lesion and does not progress further. Data shown are mean number TH+ cells in the SNc ± standard error ***p<0.001. (E) Loss of TH+ cells is accompanied by formation of α-synuclein immunopositive aggregates in lesioned animals compared to saline controls at week 1 and 3. Note the clear pattern and distribution of α-synuclein inclusions, with some surviving TH+ cells showing inclusion pathology (solid white arrows), some surviving TH+ cells without α-synuclein positive inclusions (dashed white arrows), TH-negative cells with α-synuclein positive inclusions (yellow solid arrows) and aggregates of α-synuclein in the brain parenchyma (asterisks). Images in (A, C, E) ×4 magnification, scale bar = 200µm, images in (G) ×40 magnification, scale bar = 20 µm. (TIF) [file pone.0017269.s003.tif]

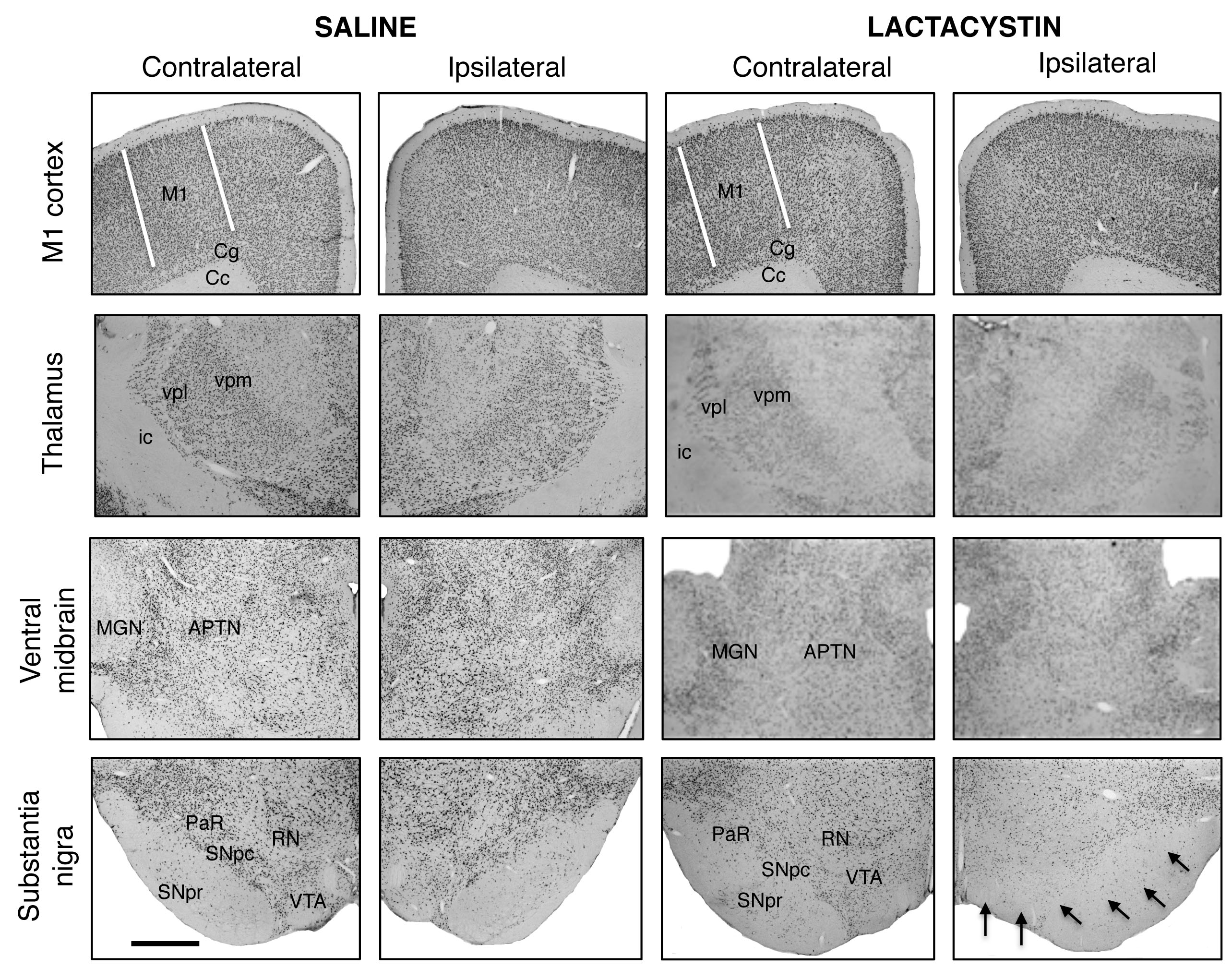

Supplement: Figure S4 — Qualitative analysis of neuronal loss in extra-nigral brain regions demonstrating MRI changes at week 1 post-lesion. (A) Lactacystin microinjection (N = 5) does not result in apparent neuronal loss in the ipsilateral primary motor (M1) cortex at this time-point. Similarly, compared to saline controls (N = 5) no neuronal loss is evident in (B) ipsilateral ventral thalamic nuclei, (C) ipsilateral ventral midbrain extra-nigral nuclei. By contrast, substantial neuronal loss is present in (D) the substantia nigra pars compacta (SNc). Note also the loss of neurons in the nearby ventral tegmental area (VTA) and substantia nigra pars reticulata (SNr). All images ×4 magnification, scale bar = 200 µm. Abbreviations: M1, primary motor cortex; cg, cingulum; cc, corpus callosum; ic, internal capsule; vpl, ventral posterolateral thalamic nucleus; vpm, ventral posteromedial thalamic nucleus; mgn, medial geniculate nucleus; APTN, anterior pretectal nucleus; PaR, pararubral nucleus; RN, red nucleus. (TIF) [file pone.0017269.s004.tif]

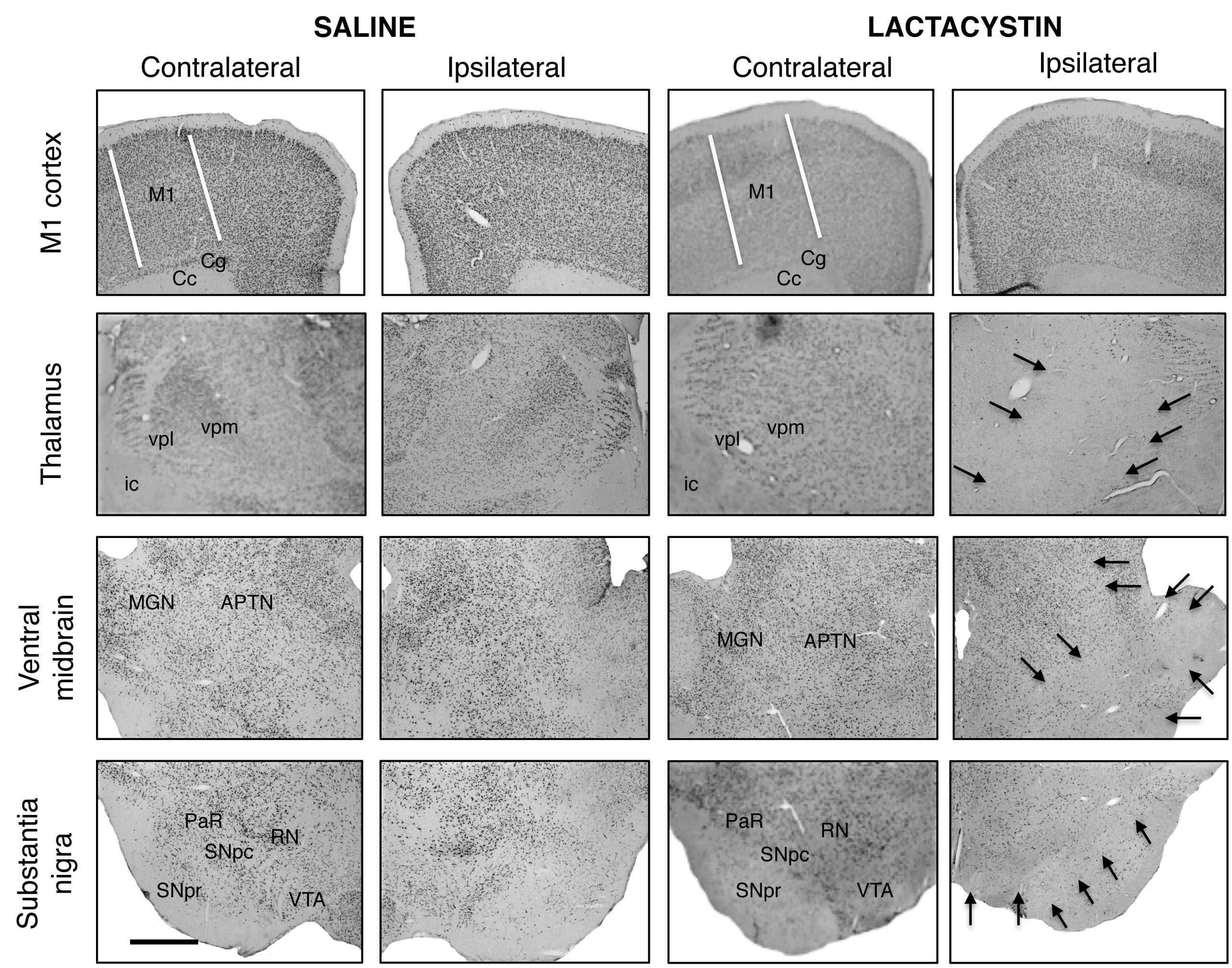

Supplement: Figure S5 — Qualitative analysis of neuronal loss in extra-nigral brain regions demonstrating MRI changes at week 3 post-lesion. (A) Lactacystin microinjection (N = 5) does not result in apparent neuronal loss in the ipsilateral primary motor (M1) cortex at this time-point. By contrast, compared to saline controls (N = 5) widespread neuronal loss is already apparent (solid black arrows) in (B) ipsilateral ventral thalamic nuclei, (C) ipsilateral ventral midbrain extra-nigral nuclei and (D) substantia nigra pars compacta (SNc). Note also the loss of neurons in the nearby ventral tegmental area (VTA) and substantia nigra pars reticulata (SNr). All images ×4 magnification, scale bar = 200 µm. Abbreviations: M1, primary motor cortex; cg, cingulum; cc, corpus callosum; ic, internal capsule; vpl, ventral posterolateral thalamic nucleus; vpm, ventral posteromedial thalamic nucleus; mgn, medial geniculate nucleus; APTN, anterior pretectal nucleus; PaR, pararubral nucleus; RN, red nucleus. (TIF) [file pone.0017269.s005.tif]
