# Supplementary material for: Evolution of Extra-Nigral Damage Predicts Behavioural Deficits in a Rat Proteasome Inhibitor Model of Parkinson's Disease
Source: PLoS One. 2011 Feb 25;6(2):e17269. doi: 10.1371/journal.pone.0017269 (PMC3045435; doi:10.1371/journal.pone.0017269)
Supplement: Table S1 — Summary of overall statistical results from repeated measures 2-way ANOVA for in vivo serial MRI measurements. (DOC) [file pone.0017269.s006.doc]

**Table S1**

| **Brain region** | **MRI measurement** | **Lesion** | **Time** | **Lesion x time** |
| --- | --- | --- | --- | --- |
| Whole brain | Volume | F(1,24)=11.70; p<0.01 | F(3,24)=0.15; ns | F(3,24)=0.97 ns |
| Ventral midbrain | Volume | F(3,57)=14.94; p<0.001 | F(3,57)=11.09; p<0.001 | F(9,57)=31.43; p<0.001 |
| Corpus striatum | Volume | F(3,54)=9.14; p<0.001 | F(3,54)=1.13; ns | F(9,54)=5.45; p<0.001 |
| Cerebral cortex | Volume | F(3,57)=24.39; p<0.001 | F(3,57)=5.65; p<0.01 | F(9,57)=15.61; p<0.001 |
| Lateral ventricles | Volume | F(3,54)=11.53; p<0.001 | F(3,54)=21.65; p<0.001 | F(9,54)=4.80; p<0.001 |
| M1 cortex | Volume | F(3,60)=19.82; p<0.001 | F(3,60)=3.68; ns | F(9,60)=1.80; ns |
| S1BF cortex | Volume | F(3,60)=11.58; p<0.001 | F(3,60)=12.11; p<0.001 | F(9,60)=2.85; ns |
| Hippocampal formation | Volume | F(3,24)=0.69; ns | F(3,24)=15.53; p<0.001 | F(9,24)=0.59; ns |
| Cerebellum | Volume | F(1,12)=0.35; ns | F(3,12)=6.917; p<0.001 | F(3,12)=0.317; ns |
| Substantia nigra | T_2_ SI ratio | F(1,21)=5.87; p<0.05 | F(3,21)=5.36; p<0.01 | F(3,21)=7.39; p<0.01 |
| Corpus striatum | T_2_ SI ratio | F(1,27)= 0.38; ns | F(3,27)=0.73; ns | F(3,27)=0.46; ns |
